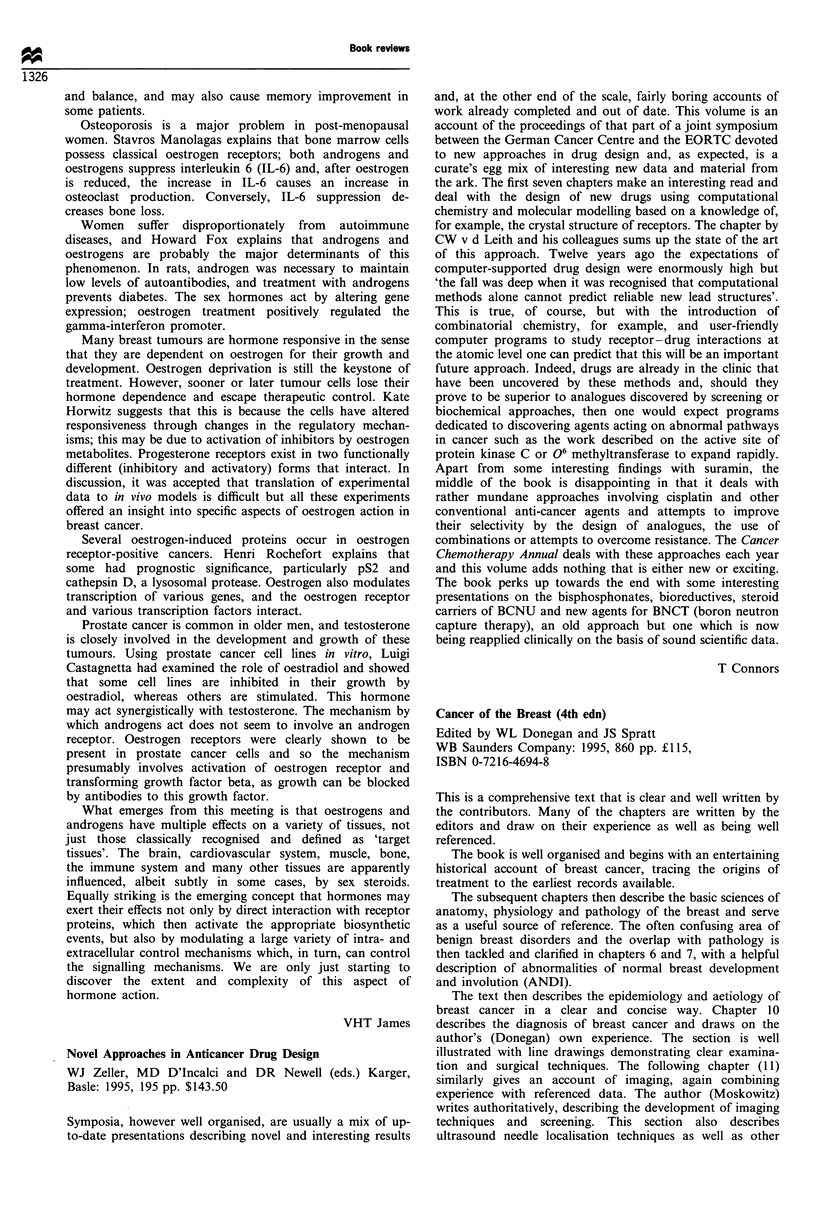# Novel approaches in anticancer drug design

**Published:** 1996-10

**Authors:** T Connors


**Novel Approaches in Anticancer Drug Design**

WJ Zeller, MD D'Incalci and DR Newell (eds.) Karger, Basle: 1995, 195 pp. $143.50

Symposia, however well organised, are usually a mix of up-to-date presentations describing novel and interesting results and, at the other end of the scale, fairly boring accounts of work already completed and out of date. This volume is an account of the proceedings of that part of a joint symposium between the German Cancer Centre and the EORTC devoted to new approaches in drug design and, as expected, is a curate's egg mix of interesting new data and material from the ark. The first seven chapters make an interesting read and deal with the design of new drugs using computational chemistry and molecular modelling based on a knowledge of, for example, the crystal structure of receptors. The chapter by CW v d Leith and his colleagues sums up the state of the art of this approach. Twelve years ago the expectations of computer-supported drug design were enormously high but 'the fall was deep when it was recognised that computational methods alone cannot predict reliable new lead structures'. This is true, of course, but with the introduction of combinatorial chemistry, for example, and user-friendly computer programs to study receptor–drug interactions at the atomic level one can predict that this will be an important future approach. Indeed, drugs are already in the clinic that have been uncovered by these methods and, should they prove to be superior to analogues discovered by screening or biochemical approaches, then one would expect programs dedicated to discovering agents acting on abnormal pathways in cancer such as the work described on the active site of protein kinase C or $O^6$ methyltransferase to expand rapidly. Apart from some interesting findings with suramin, the middle of the book is disappointing in that it deals with rather mundane approaches involving cisplatin and other conventional anti-cancer agents and attempts to improve their selectivity by the design of analogues, the use of combinations or attempts to overcome resistance. The *Cancer Chemotherapy Annual* deals with these approaches each year and this volume adds nothing that is either new or exciting. The book perks up towards the end with some interesting presentations on the bisphosphonates, bioreductives, steroid carriers of BCNU and new agents for BNCT (boron neutron capture therapy), an old approach but one which is now being reapplied clinically on the basis of sound scientific data.

T Connors